# Effects of Herbal Supplementation on Growth Performance of Kenguri Sheep Exposed to Heat Stress

**DOI:** 10.3390/ani15091285

**Published:** 2025-04-30

**Authors:** Ebenezer Binuni Rebez, Chinnasamy Devaraj, Jacob Ninan, Mullakkalparambil Velayudhan Silpa, Shanmugam Venkatesa Perumal, Artabandhu Sahoo, Frank Rowland Dunshea, Veerasamy Sejian

**Affiliations:** 1 Centre for Climate Resilient Animal Adaptation Studies, ICAR-National Institute of Animal Nutrition and Physiology, Adugodi, Bangalore 560030, India; binunirebez.e@gmail.com (E.B.R.); drcdeva@gmail.com (C.D.); sahooarta1@gmail.com (A.S.); 2 Rajiv Gandhi Institute of Veterinary Education and Research, Kurumbapet, Puducherry 605009, India; ninanjacob@river.edu.in (J.N.); mv.silpa@gmail.com (M.V.S.); sbvenky4@gmail.com (S.V.P.); 3 School of Agriculture, Food and Ecosystem Sciences, Faculty of Science, The University of Melbourne, Parkville, Melbourne, VIC 3010, Australia; 4 School of Biology, Faculty of Biological Science, The University of Leeds, Leeds LS2 9JT, UK

**Keywords:** climate resilience, growth, heat stress, herbal supplement, sheep

## Abstract

A study was designed to explore the possibility of using herbal supplementation to sustain growth performance during heat stress exposure in Kenguri sheep. This 60-day study was conducted on 24 Kenguri ewes (1–2 years old), randomly assigned to four treatment groups (*n* = 6 per group) as follows: KC (*n* = 6; Kenguri Control), KHS (*n* = 6; Kenguri Heat Stress), KCS (*n* = 6; Kenguri Control and herbal supplement), and KHSS (*n* = 6; Kenguri Heat Stress and herbal supplement). The herbal mixture of Ocimum sanctum (Tulsi), Emblica officinalis (Amla), Morinda citrifolia (Noni), Withania somnifera (Ashwagandha), and Phyllostachys edulis (Bamboo) was used in this study. The herbal supplement used in the present study was given to the KCS and KHSS groups’ animals in dry powder form at a dose of 0.8 g/Kg BW/Day. All variables were recorded fortnightly, and gene expression analysis was performed at the end of the experiment. The results indicated that the recorded temperature–humidity index (THI) provided thermal comfort for KC and KCS while inducing extremely severe heat stress to the KHS and KHSS groups. Heat stress did not alter the feed intake, while the herbal supplement during heat stress increased the feed intake from day 30 onwards. Furthermore, heat stress significantly (*p* < 0.001) increased the water intake, while the herbal supplement did not alter the heat stress-induced water intake. In addition, neither heat stress nor herbal supplements influenced the body weight and allometric measurements studied. Furthermore, heat stress significantly (*p* < 0.01) decreased the level of plasma tri-iodo-thyronine (T_3_) and thyroxin (T_4_) and had a non-significant effect on plasma growth hormone (GH), insulin-like growth factor-1 (IGF-1), while the herbal supplements significantly (*p* < 0.01) increased the levels of all these hormones studied. Likewise, in peripheral blood mononuclear cells (PBMCs) the expression patterns of *growth hormone receptor (GHR), Insulin-like growth factor 1 (IGF1)* and *prolactin receptor* (*PRLR)* were significantly (*p* < 0.001) downregulated during heat stress (0.25, 0.3, and 0.48-fold change, respectively). However, the herbal supplement significantly (*p* < 0.01) increased the heat stress-induced reduction in the expression pattern of these three genes (0.65, 0.61, and 0.61-fold change, respectively). Therefore, from this study, it could be concluded that although the herbal supplements did not bring positive changes in body weight and allometric measurements, it still had a beneficial impact on the endocrinology and genes governing growth performance in Kenguri ewes. Thus, the herbal feed additive used in the study shows promise for relieving heat stress in Kenguri ewes.

## Simple Summary

Sheep husbandry has played a crucial role in supporting rural livelihoods. However, climate change associated heat stress in sheep has a detrimental impact on growth performance. In order to address the negative effects of heat stress on animal growth, scientists have been exploring nutritional solutions like herbal supplements as they have significant effects on feed intake, water intake, and hormonal and molecular mechanisms governing growth in major livestock. However, negligible studies have delved into these aspects in heat-stressed sheep. Thus, the study targeted addressing these concerns focusing on the native Indian sheep breed, Kenguri. The 60-day study conducted using 24 ewes was divided into four groups under different treatments in climate-controlled chambers, including herbal supplementation. The results indicated that herbal supplements improved feed intake and growth-related hormones in heat-stressed ewes. Further, the herbal supplement had a significant role in increasing the expression pattern of genes governing growth. The study established that herbal supplements support the growth performance of heat-stressed sheep. The conclusions show promise for improving climate resilience in Kenguri ewes and open the way for navigating future strategies that sustain sheep production through innovative nutrition and breeding strategies.

## 1. Introduction

Sheep farming has long been a sustainable source of income for rural communities [1]. The global sheep population has increased, reaching 1.266 billion in 2021, with China being the country with the largest sheep numbers [2]. Recently, there has been a greater understanding of the detrimental impacts of heat stress associated with climate change on livestock production [3,4,5,6]. As the climate crisis intensifies, urgent conservation efforts are needed for livestock species [7]. The conservation and production of indigenous, thermo-tolerant breeds can mitigate the climate change impact on the availability of animal produce globally [7,8], demanding significant research efforts for screening the climate resilience potential of native sheep breeds.

As a voluntary adaptive response to increased ambient temperature conditions, sheep decrease feed intake and increase water intake. Further, the reduced feed intake associated with heat stress affects animals particularly at various productive stages, reflecting reduced growth performance [7]. Elevated temperature also causes changes in their biological functions, like altered endocrine dynamics, affecting the growth performance of sheep. Heat stress in sheep exhibits a discernible effect on growth performance by causing decreased anabolic activity due to reduced feed intake and increased tissue catabolism [9,10,11,12]. The restriction of growth and other production variables’ performance during heat stress is attributed to stress hormones, particularly corticosteroids [13]. Heat stress also reduces the secretion of thyroid hormones (triiodothyronine and thyroxine), resulting in altered thermogenesis and basal metabolism, which can negatively influence the productive traits like growth and fattening in animals [13,14]. The expression patterns of genes that encode growth hormone (GH), growth hormone receptor (GHR), insulin-like growth factor-1 (IGF-1), leptin (LEP), leptin receptor (LEPR), and thyroid hormone receptor (THR) are linked to the impacts of heat stress on growth performance in small ruminants [15]. Apart from hormonal changes reflecting growth potential, the expression patterns of genes have also been used as potential indicators to evaluate the growth performance of heat-stressed sheep. However, there is a paucity of validated growth production-related biomarkers for assessing heat stress impacts in sheep.

Herbal supplements have been widely used to prevent and treat diseases in both human and veterinary medicine due to their no-residue nature, low toxicity, and lack of adverse effects [16]. In addition, it has been established that herbal mixtures improve feed intake, immunity, and performance and decrease disease occurrence during heat stress in cattle [17,18], buffaloes [19,20], sheep [16,21,22], goats [23,24], pigs [25], poultry [26,27,28,29], donkeys [30], and mice [31,32]. The herbal mixtures have anti-inflammatory, antioxidant, and anti-microbial effects [33,34], making them an ideal nutritional supplement for heat-stressed animals. The complex pathogenesis of heat stress in animals often means a single herbal remedy has an insignificant effect and frequently does not have the potential effect to ameliorate heat stress; instead, a combination of herbs would help to improve the overall impact of herbal components on heat-stressed cells [35]. Herbs such as Tulsi, Amla, Noni, Ashwagandha, and Bamboo have established roles in improving feed intake, growth performance, and immunity in addition to their antioxidant, analgesic, hepatoprotective, cardioprotective, and adaptogenic properties [16,19,20,23,26].

Based on the available literature, it is evident that there is no detailed study on the influence of herbal additives on the growth performance, endocrinology, and molecular mechanisms governing growth in sheep during heat stress exposure. Thus, a hypothesis was framed based on a published study unravelling the significance of a herbal mixture in promoting growth performance in heat-stressed sheep [36]. Kenguri sheep, being a popular breed with high potential for meat production, was the breed of choice for the study. The objective of the present work was to study the behavioural responses (feed intake and water intake) of heat-stressed and herbal-supplemented Kenguri sheep. Further, the study attempted to understand the influence of the herbal supplement on the growth performance of heat-stressed indigenous Kenguri sheep using endocrine- and genetic growth performance-related biomarkers.

The present study was the first to evaluate the beneficial effects of herbal supplements on growth performance in heat-stressed Kenguri sheep through integration of behavioural, hormonal, and genetic variables, providing valuable insights on the use of herbal supplements. This study’s novel findings can contribute to the development of nutritional strategies and refinement of breeding programs for improving sheep resilience in challenging climatic conditions.

## 2. Materials and Methods

### 2.1. Experimental Animals

Kenguri, or Tenguri, is a well-known indigenous sheep breed popular for mutton production in the north-eastern region of Karnataka state, which features a tropical climate. The current work used 24 adult, one-to-two-year-old female Kenguri sheep with a body weight ranging between 15 and 23 kg. The grouping of animals was done randomly so that the average body weights of all four groups’ ewes were not statistically different at the start of the study. The animals were acclimatised to the study location for one month. During this period, the animals were exposed to the experimental diet and the local environmental conditions to acclimatise them before the start of the study. The study animals were housed in well-ventilated barns with asbestos roofing and were provided with a high-quality diet and drinking water. The feed and water residues were collected at fortnightly intervals to calculate the feed intake and water intake, respectively. Prophylactic measures (vaccination and deworming) against common infectious diseases and endo- and ectoparasitic infestations were performed according to the health management plan for maintaining animal health throughout the experimental period.

### 2.2. Experimental Location, Climate, and Design

The experiment was conducted at the Centre for Climate Resilient Animal Adaptation Studies (CCRAAS), ICAR-National Institute of Animal Nutrition and Physiology (ICAR-NIANP), Bangalore, India. The experimental location lies in the country’s southern Deccan plateau at longitude 12°57′04.3′′ N, latitude 77°36′25.3′′ E, and an elevation of 920 m above mean sea level (MSL). The study was carried out between the months of June and August. The experiment was conducted in the year 2024. Table 1 presents the average weather variables and temperature–humidity index (THI) recorded at 8:00 and 14:00 in the thermoneutral and heat stress chambers. The average THIs in the thermoneutral zone (TNZ) chamber from 8:00 h to 14:00 h were 71.25 and 68.93, respectively. Additionally, for the heating chamber, the average THIs from 8:00 h to 14:00 h were 72.27 and 93.34, respectively. Further, the average THIs from 16.00 h to 10.00 h were 74.38 and 74.45, respectively, in the TNZ and heating chamber, with negligible difference between the chambers. The animals in the thermoneutral zone chamber are exposed to a range of temperatures wherein they can maintain a stable body temperature without expending extra energy.

The THI was calculated using McDowell, ref. [37]’s formula, *THI = 0.72 (Tdb + Twb) + 40.6, incorporating DBT and WBT*. The THI obtained during the morning and afternoon clearly indicated the significantly (*p* < 0.01) higher THI obtained at 14.00 in the heat stress chamber as compared to the thermoneutral chamber. As per McDowell [37]’s model, THI values of 72 and less are considered comfortable; THI values between 75 and 78 are considered stressful; and THI values above 78 are considered extreme distress. The average THI value of 93.34 obtained at 14.00 clearly demonstrated the extremely severe heat stress experienced by both the KHS and KHSS groups. Further, the THI of 68.93 obtained in the thermoneutral chamber clearly indicated the comfortable condition experienced by the KC and KCS groups.

This 60-day study in a climate-controlled facility was conducted on 24 Kenguri ewes (1–2 years old). The body weight was taken as a criterion for grouping these animals. The animals were randomly allocated into four groups so that the average body weight across these groups was not statistically significant at the start of the study. The groupings were done based on a 2 × 2 factorial design that took into account temperature and diet as independent factors. Accordingly, two groups were placed in a thermoneutral chamber with different nutritional regimes, while the other two groups were kept in the heating chamber with two different dietary regimes. Thus, the groups were named as follows: KC (*n* = 6; Kenguri Control; standard diet), KHS (*n* = 6; Kenguri Heat Stress; standard diet), KCS (*n* = 6; Kenguri Control Supplement; standard diet + herbal powder), and KHSS (*n* = 6; Kenguri Heat Stress Supplement; standard diet + herbal powder). This sample size of six animals in each group is a standard practice for maintaining a minimum number of animals in each group for this study design. Following the same criteria, four animals with similar body weights were chosen and distributed, with one animal to each group. Likewise, the same process was repeated to allocate the same five animals to different groups, accordingly. Thus, the researchers were aware of the animals allocated to each group. All of the 24 animals chosen for this study were healthy, without ill-health. Those animals that were not healthy or had sicknesses were excluded from the study. The best 24 animals in the flock were chosen for this study.

In both chambers, the animals were individually housed in a pen with individual feeding and watering facilities and floor space to ensure free movement. The individual pens were kept on an elevated platform, and the floor material was plastic. The individual animals were housed in the particular pen throughout the study period. The diet comprised a 60% roughage (Hybrid Napier) and a 40% concentrate mixture. The experimental animals were fed in their respective enclosures. Table 1 lists the ingredients and chemical composition of the concentrate and roughage feed provided for the experimental animals. Table 2 details the composition of the dietary herbal supplement, and Table 3 lists the composition of herbal supplements used in the study. The herbal supplement was composed of a mixture of *Ocimum sanctum* (Tulsi), *Emblica officinalis* (Amla), *Morinda citrifolia* (Noni), *Withania somnifera* (Ashwagandha), and *Phyllostachys edulis* (Bamboo). The herbal supplement used in the present study was given to the KCS and KHSS groups’ animals in dry powder form at a dose of 0.8 g/Kg BW/Day along with the concentrate ration.

The entire herbal composition, their proportions, and the dose rate were calculated based on a review of the published literature. Further, all five ingredients were procured in powder form and the appropriate proportions of each ingredient were uniformly mixed, as described in Table 3, to prepare 100 g of the herbal mixture. The final dose rate of 0.8 g/Kg BW/Day herbal supplements has the same proportion of the extracts of these herbal plants, as indicated in Table 3. In addition, we would like to clarify that we did not measure the active ingredients. First, we wanted to try the crude extract of these plants to establish their heat stress-ameliorating effects. In subsequent studies, the active ingredients will be established.

**Figure 1:**
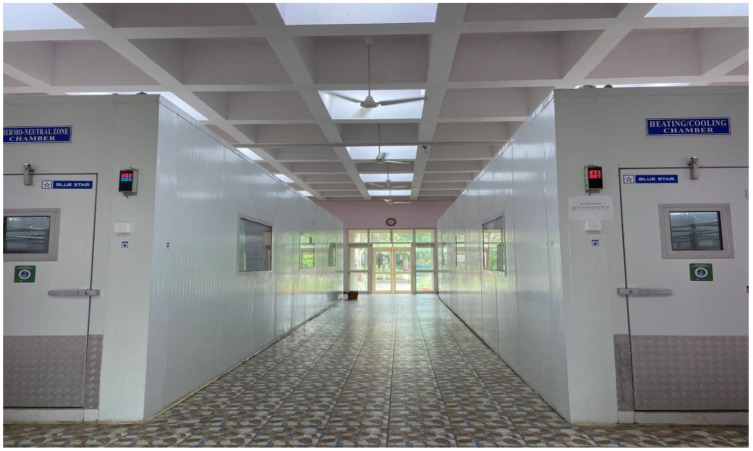
Climate chambers: the thermoneutral zone chamber (**left**) and the heating/cooling chamber (**right**)’s outside views.

**Figure 2:**
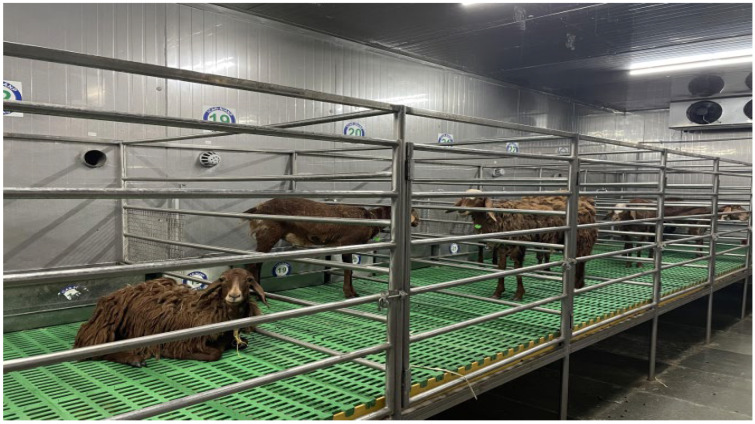
Kenguri sheep kept inside the climate chamber.

The climate chamber measures 12.8 m long, 3.41 m wide, and 2.87 m high, with a built area of 143.00 m^2^, and is made of laminated steel sheets with anti-corrosion protection and filled with polystyrene foam. Each chamber has a holding capacity of 12 animals with individual animal pen areas of 1.95 m^2^. Figure 1 describes the outside view of the climate chambers. The pens were partitioned with stainless steel rods to prevent the entry of sheep to adjacent pens. At the same time, this ensured that the animals were free of socialising stress since they could see other animals. The floor of each individual pen was 0.6 m above the floor of the climate chamber. Each pen had an individual feeder, waterer, and urine and faecal collection trays. The animals were maintained in individual pens throughout the study duration. Figure 2 describes the Kenguri sheep kept inside the climate chambers. Both the thermal comfort zone climate chamber and heating climate chamber are designed to provide the appropriate environmental conditions required for any environmental stress-related studies. It is an established fact that the thermal comfort zone for sheep ranges between 20 and 28 °C [38]. Therefore, the thermal comfort zone chamber was maintained at 24 °C throughout the study period for a six hour duration daily, between 10:00 h and 16:00 h, to provide the ideal thermal comfort zone to the KC and KCS groups’ ewes. In the heating climate chamber, the KHS and KHSS groups’ ewes were exposed to a simulated heat stress model, as described [39]. The simulated heat stress model was developed based on thirty years of ambient temperature and relative humidity data for the summer season (April, May, and June) in the study locality [39]. The model was developed with the intention of mimicking the natural environmental stress condition in the climate chamber. The time duration of six hours, between 10:00 h and 16:00 h, was chosen since only during this period were the animals exposed to heat stress while grazing in an extensive system of rearing. Moreover, from an animal ethical point of view, the simulated heat stress model was more practical and less stressful than constant heat stress models. The KC and KCS animals were exposed to 24 °C in the thermoneutral zone chamber to maintain thermal comfort, while the KHS and KHSS groups’ animals were exposed to heat stress in the heating chamber under a simulated heat stress model [39].

The study animals in the thermoneutral zone chamber and heating chamber were exposed to the respective temperatures for a duration of six hours a day (between 10:00 to 16:00) during the experimental period. Feeding and watering of the experimental animals were carried out in their individual cabins. All the procedures involved in the study, including the subjection of experimental animals to heat stress, were carried out with the approval of the institute’s animal ethics committee, letter number V-11011(13)/14/2021-CPCSEA-DADF.

### 2.3. Blood Collection for Hormonal Analysis

Blood sampling for hormonal analysis was performed at fortnightly intervals at 11:00 h. Blood samples (4 mL) were collected from the external jugular vein of the animals in all four groups using heparin-coated vacutainers. The plasma was harvested immediately following the blood collection by subjecting the blood samples to centrifugation at 3500 rpm for 20 min. The separated plasma samples were stored at −20 °C until hormonal analysis.

The enzyme-linked immunosorbent assay (ELISA) method was used to estimate the level of the target hormones in the sample (Puregene kit, Genetix, New Delhi). The reagents, standard solution, and samples were prepared as instructed and were thawed before using them in the reaction. Additionally, all the reagents were mixed well without foaming. Once the procedure started, all steps were completed without interruption. To the standard well, 50 μL of standard solution were added. Further, to the sample wells, 40 μL of the sample and then 10 μL of anti-GH antibody were added. Following this, 50 μL of streptavidin–HRP were added to the sample and standard wells (except the blank control well). The plates were mixed gently and sealed to incubate at 37 °C. The sealer was removed, and the plates were washed approximately 5–6 times using the prepared wash buffer (400 μL/well for each wash). Then, the plates were blotted by sharply striking against the absorbent paper to remove residual droplets (done by hand 6 times). Then, 50 μL of substrate solution A, followed by an equal amount of substrate solution B, were pipetted into each well at timed intervals. The plates were covered with a sealer and incubated for 10 min at 37 °C in the dark. Following this, upon the addition of 50 μL of stop solution to all the wells, the colour change from blue to yellow was observed, and the optical density (OD) value was determined using a microplate reader set to 450 nm within 10 min after the stop solution was added. The ODs of unknown samples were read against the standard curve drawn using immunoassay software and analysed.

### 2.4. Blood Collection for PBMC Isolation

On the last day of the study, blood sampling for PBMC isolation was carried out at 11.00 h simultaneously from all the study animals. Five ml of blood samples were collected from the external jugular vein in EDTA-containing vacutainers and were stored in ice. The blood samples were processed immediately for PBMC isolation. To the samples, ice-cold 1× RBC lysis buffer was added, and this was incubated at room temperature for 10 min, followed by centrifugation at 2500 rpm at 4 °C for 5 min. The total RNA was isolated from the PBMCs using the GeneJET Whole Blood RNA Purification Mini Kit (Thermo Scientific, Vilnius, Lithuania) in accordance with the manufacturer’s instructions. Fresh nuclease-free micro-centrifuge tubes were used to collect the eluted RNA.

The Revert Aid First Strand cDNA Synthesis Kit (Thermo Scientific, Vilnius, Lithuania) was used to reverse-transcribe the total RNA into complementary DNA (cDNA), following the manufacturer’s protocol, for use in real-time quantitative polymerase chain reaction (RT-qPCR). The product of the first-strand cDNA synthesis was diluted to a final concentration of 25 ng/μL with nuclease free water (NFW), and 2 μL of diluted cDNA were used for each reaction in qPCR. An online NCBI primer design software was used to create a gene-specific primer, and its specificity was verified using BLAST and Primer3 (http://www.ncbi.nlm.nih.gov/tools/primer-blast/ accessed on 15 March 2024). Table 4 describes the primer sequences of the housekeeping gene and target genes.

The relative expressions of the targeted genes were studied using SYBR Green chemistry (Maxima SYBR Green qPCR master mix, Fermentas, Waltham, MA, USA). The 20 μL reaction was carried out in triplicate using 50 ng of template and 0.5 μM primer concentrations. The reaction condition was enzyme activation at 95 °C for 10 min and an amplification cycle (40 cycles; initial denaturation at 95 °C for 15 s, annealing at 60 °C for 30 s, and extension at 72 °C for 30 s). By examining the melt curve analysis towards the end of the reaction, non-specific amplification was ruled out. As an internal control, the housekeeping gene *GAPDH* (glyceraldehyde 3-phosphate dehydrogenase) was used to compare the relative expression of target genes using the 2^−ΔΔCT^ method.

### 2.5. Variables Studied

The targeted weather variables in the study were as follows: maximum temperature, minimum temperature, and relative humidity, which were recorded using a thermo-hygrometer, and the dry-bulb temperature and wet-bulb temperature, which were recorded using a dry-bulb and wet-bulb thermometer, respectively. The weather variables were recorded twice daily at 8:00 h and 14:00 h. The THI was calculated using McDowell’s 1972 model [37]. Feed intake (FI) (g/w^0.75^/day) and water intake (WI) (L/KgDMI/day) were calculated fortnightly based on the feed and water residue data. The feed and water residue data were recorded daily at 7.00 h, and their fortnightly average was taken to maintain similarity with other parameters recorded in the study. The individual feed and water troughs provided for all 24 ewes facilitated the recording of the feed and water residue data, respectively. The feed and water residue data were measured using a digital weighing balance and measuring cylinder, respectively. The FI/WI was calculated using the following formula: feed/water offered on the previous day-feed/water left in the trough. The body weight of the animals was recorded at 7.00 h using a digital weighing balance, and allometric measurements were recorded at 7.00 h using a measuring tape at fortnightly intervals. Blood sampling for hormonal analysis was carried out at 11.00 h at fortnightly intervals, and hormones, including growth hormone (GH), insulin-like growth factor-1 (IGF-1), thyroxine (T4), and triiodothyronine (T3), were estimated by employing a Puregene enzyme-linked immunoassay (ELISA) kit and using a microplate reader. The collection of blood samples for the gene expression study was carried out at the end of the trial. The targeted genes were *IGF1*, growth hormone receptor (*GHR*), prolactin receptor (*PRLR*), and thyroid hormone receptor (*THR*).

### 2.6. Data Analysis

The feed intake, water intake, body weight, allometric measurements, and endocrine variables in the study were subjected to 2 × 2 factorial analysis with temperature, diet, and experimental days as fixed effects and the animal as a random effect. Furthermore, the interaction effects between diet × temperature, diet × experimental day, temperature × experimental day, and diet × temperature × experimental day were all considered. The significance of each effect was assessed by least square analysis (LSD). Similarly, the changes in relative expression of the PBMC target genes with *GAPDH* as the housekeeping gene were analysed by 2 × 2 factorial analysis with temperature, diet, and temperature × diet as fixed factors and the animal as a random effect. The significant difference between the main effects was compared using Bonferroni values. The significance level was set at *p* < 0.05. The data obtained were statistically analysed using the SPSS software (Version 29.0), considering the Restricted Maximum Likelihood (REML) approach.

## 3. Results

### 3.1. Effect of Heat Stress and Herbal Supplementation on Feed Intake and Water Intake in Kenguri Sheep

The feed and water intake were observed to be significantly influenced by several experimental factors, as depicted in Table 5.

#### 3.1.1. Results on Feed Intake

Feed intake increased under heat stress (49.93 vs. 51.30 g/w0.75/day for thermoneutral vs. heat stress; *p* < 0.01) and was further enhanced by herbal supplementation (50.03 vs. 51.20 g/w0.75/day for control vs. herbal diet; *p* < 0.05) (Table 5).

In addition, the feed intake was different between the days, being lowest on day 15 and highest on day 30 (50.61, 48.95, 51.55, 50.54, and 51.43 g/w^0.75^/day for day 0, day 15, day 30, day 45, and day 60, respectively, *p* < 0.01). Among the various interactions, temperature × day (*p* < 0.001), diet x day (*p* < 0.01), and temperature x diet x day (*p* < 0.01) influenced the feed intake.

#### 3.1.2. Results on Water Intake

The water intake significantly increased during heat stress (4.45 vs. 6.51 L/Kg DMI/day for thermoneutral and heat stress conditions, respectively, *p* < 0.001). However, the diet did not influence the water intake (5.32 vs. 5.63 L/Kg DMI/day for the control and herbal diet, respectively, *p* = 0.481) (Table 5). In addition, the water intake was different between the days, being lowest on day 0 and highest on day 45 (3.44, 6.13, 5.95, 6.17, and 5.70 L/Kg DMI/day for day 0, day 15, day 30, day 45, and day 60, respectively, *p* < 0.001). Among the various interactions studied, only temperature × day significantly increased the water intake (*p* < 0.001). However, temperature × diet (*p* = 0.55), diet × day (*p* = 0.14), and temperature × diet × day (*p* = 0.69) did not influence the water intake.

### 3.2. Effect of Heat Stress and Herbal Supplementation on Body Weight in Kenguri Sheep 

Both temperature treatment (20.83 vs. 20.93 kg for thermoneutral and heat stress conditions, respectively, *p* = 0.89) and diet (20.64 vs. 21.12 kg for control and herbal diet, respectively, *p* = 0.56) did not influence body weight (Table 6).

However, the experimental days highly significantly (*p* < 0.001) influenced the body weight, with the lowest value recorded on day 0 and the highest on day 60 (18.97, 20.00, 20.80, 21.75, and 22.90 kg for day 0, day 30, day 45, and day 60, respectively, *p* < 0.001). Among the various interactions, only diet × day significantly (*p* < 0.001) influenced the body weight. However, temperature × diet (*p* = 0.48), temperature × day (*p* = 0.69), and temperature × diet × day (*p* = 0.29) interactions did not alter the body weight in the study.

### 3.3. Effect of Heat Stress and Herbal Supplementation on Allometric Measurements in Kenguri Sheep

The study revealed both temperature and diet to have a comparable impact (*p* > 0.05) on the allometric measurements body length, body height, and chest girth (Table 7). However, the experimental days highly influenced all the allometric variables, with the highest recording observed on day 60. Among the various interactions, only diet × day significantly (*p* < 0.05) influenced body length. However, no other significant interactions were observed for the other allometric variables.

### 3.4. Effect of Heat Stress and Herbal Supplementation on Endocrine Variables in Kenguri Sheep

The experimental factors (diet, temperature, day, and the interaction effects) were observed to significantly influence the endocrine profile in Kenguri sheep (Table 8).

#### 3.4.1. Growth Hormone (GH)

Heat stress did not influence the plasma growth hormone (GH) concentration (3.28 vs. 2.85 ng/mL for thermoneutral and heat stress conditions, respectively, *p* = 0.33), however, the herbal supplement significantly (*p* < 0.01) increased the GH concentration (2.32 vs. 3.80 ng/mL, for control and herbal diet, respectively, *p* < 0.01) (Table 8).

Further, the GH concentration was different between the days, with the lowest value recorded on day 0 and the highest value recorded on day 60 (2.79, 2.67, 3.13, 3.22, and 3.50 ng/mL for day 0, day 30, day 45, and day 60, respectively, *p* < 0.001). Among the various interactions, only diet x day significantly (*p* < 0.001) influenced the GH levels. However, temperature x diet (*p* = 0.29), temperature × day (*p* = 0.49), and temperature × diet × day (*p* = 0.76) interactions did not influence the GH concentration in the study.

#### 3.4.2. Insulin-like Growth Factor (IGF-1)

Heat stress did not influence the plasma insulin-like growth factor (IGF-1) concentration (55.77 vs. 50.25 ng/mL for thermoneutral and heat stress conditions, respectively, *p* = 0.30), however, the herbal supplement significantly increased the IGF-1 concentration (45.63 vs. 60.38 ng/mL, for control and herbal diet, respectively, *p* < 0.01) (Table 8). Further, the IGF-1 concentration was different between the days, with the lowest value recorded on day 0 and the highest value recorded on day 60 (48.63, 48.71, 54.76, 56.45, and 56.48 ng/mL for day 0, day 30, day 45, and day 60, respectively, *p* < 0.01). Among the various interactions, only diet × day significantly (*p* < 0.001) influenced the IGF-1 levels. However, temperature × diet (*p* = 0.45), temperature × day (*p* = 0.07), and temperature × diet × day (*p* = 0.42) interactions did not influence the IGF-1 concentration in the study.

#### 3.4.3. Tri-Iodo-Thyronine (T3)

The plasma T3 concentration significantly decreased during heat stress (28.77 vs. 24.79 nmol/L for thermoneutral and heat stress conditions, respectively, *p* < 0.001) and was significantly increased by the herbal supplement (22.74 vs. 30.82 nmol/L for control and herbal diet, respectively, *p* < 0.001) (Table 8). However, the experimental day did not influence the T3 concentration. Further, temperature × diet, diet × day, temperature × day, and temperature × diet × day interactions highly significantly (*p* < 0.001) influenced the plasma T3 concentration.

#### 3.4.4. Thyroxine (T4)

The plasma T4 concentration significantly decreased during heat stress (118.78 vs. 101.70 nmol/L for thermoneutral and heat stress conditions, respectively, *p* < 0.05) and was significantly increased by the herbal supplement (92.76 vs. 127.71 for control and herbal diet, respectively, *p* < 0.001) (Table 8). Further, the plasma T4 concentration was different between the days, with the lowest value on day 0 and the highest value on day 60 (98.0, 105.86, 108.42, 117.62, and 121.28 for day 0, day 15, day 30, day 45, and day 60, respectively, *p* < 0.001). In addition, the temperature × diet (*p* < 0.05), temperature × day (*p* < 0.001), diet × day (*p* < 0.001), and temperature × diet × day interactions influenced the plasma T4 concentration.

### 3.5. Effect of Heat Stress and Herbal Supplementation on Expression Pattern of Targeted Genes in Kenguri Sheep

#### 3.5.1. Expression Pattern of GHR

**Figure 3:**
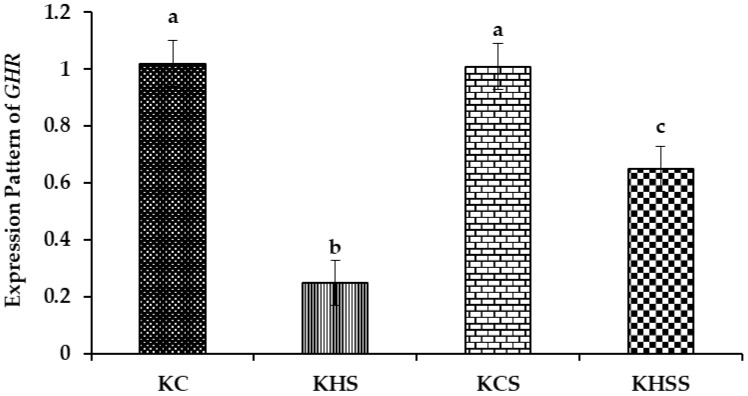
Effect of heat stress and herbal supplementation on the GHR mRNA expression pattern in Kenguri sheep. The expression patterns of *GHR* in the KC, KHS, KCS, and KHSS groups were 1.02 ± 0.08, 0.25 ± 0.08, 1.01 ± 0.08, and 0.65 ± 0.08, respectively. KC-Kenguri Control; KHS-Kenguri Heat Stress; KCS-Kenguri Control Supplement; KHSS- Kenguri Heat Stress Supplement. Different superscript letters (a, b and c) indicated extremely significant statistical differences among the treatment groups (*p* < 0.001).

The effect of heat stress and herbal supplementation on the *growth hormone receptor (GHR)* expression pattern in Kenguri sheep is described in Figure 3.

Among the different groups, significantly (*p* < 0.05) higher expression of *GHR* was recorded in the KC and KCS groups, followed by the KHS and KHSS groups. The expression of *GHR* was significantly reduced by heat stress (1.02 vs. 0.25; *p* < 0.001), but this significant reduction was increased by supplementation with herbs (0.25 vs. 0.65; *p* < 0.05). Further, there was a significant (*p* < 0.05) interaction between heat treatment and diet, such that the herbal supplementation increased the *GHR* expression during heat stress (0.25 vs. 0.65) but not under thermoneutral conditions (1.02 vs. 1.01).

#### 3.5.2. Expression Pattern of IGF1

**Figure 4:**
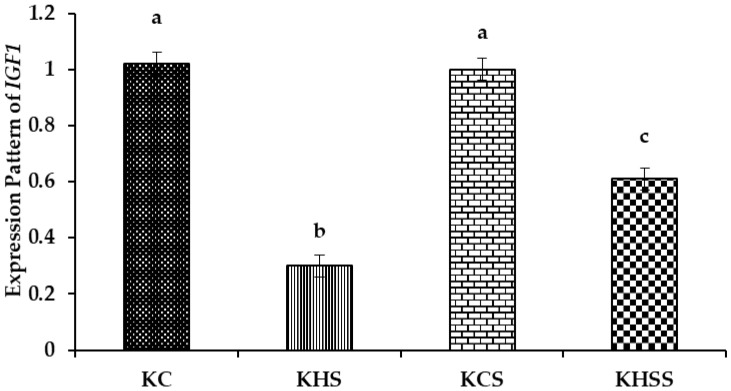
Effect of heat stress and herbal supplementation on the IGF1 mRNA expression pattern in Kenguri sheep. The expression patterns of *IGF1* in the KC, KHS, KCS, and KHSS groups were 1.02 ± 0.04, 0.3 ± 0.04, 1.0 ± 0.04, and 0.61 ± 0.04, respectively. KC-Kenguri Control; KHS-Kenguri Heat Stress; KCS-Kenguri Control Supplement; KHSS- Kenguri Heat Stress Supplement. Different superscript letters (a, b and c) indicated extremely significant statistical differences among the treatment groups (*p* < 0.001).

The effect of heat stress and herbal supplementation on the insulin-like growth factor (*IGF1*) expression pattern in Kenguri sheep is described in Figure 4.

Among the different groups, significantly (*p* < 0.001) higher expression of *IGF1* was recorded in the KC and KCS groups, followed by the KHS and KHSS groups. The expression of *IGF1* was significantly (*p* < 0.001) reduced by heat stress (1.02 vs. 0.3), but this significant reduction was increased by supplementation with herbs (0.30 vs. 0.61; *p* < 0.001). Further, there was a significant (*p* < 0.01) interaction between heat treatment and diet, such that the herbal supplementation increased the *IGF1* expression during heat stress (0.30 vs. 0.61) but not under thermoneutral conditions (1.02 vs. 1.00).

#### 3.5.3. Expression Pattern of PRLR

**Figure 5:**
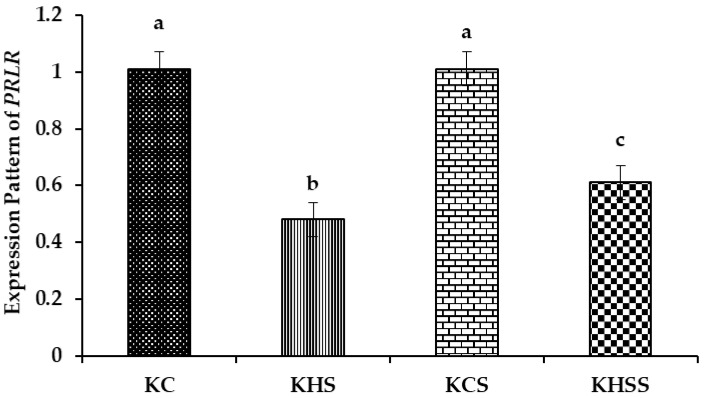
Effect of heat stress and herbal supplementation on the PRLR mRNA expression pattern in Kenguri sheep. The expression patterns of *PRLR* in the KC, KHS, KCS, and KHSS groups were 1.01 ± 0.06, 0.48 ± 0.06, 1.01 ± 0.06, and 0.61 ± 0.06, respectively. KC-Kenguri Control; KHS-Kenguri Heat Stress; KCS-Kenguri Control Supplement; KHSS- Kenguri Heat Stress Supplement. Different superscript letters (a, b and c) indicated extremely significant statistical differences among the treatment groups (*p* < 0.001).

The effect of heat stress and herbal supplementation on the *prolactin receptor* (*PRLR*) expression pattern in Kenguri sheep is described in Figure 5.

Among the different groups, significantly (*p* < 0.001) higher expression of *PRLR* was recorded in the KC and KCS groups, and the lowest was recorded in the KHS and KHSS groups. The expression of *PRLR* was significantly (*p* < 0.001) reduced by heat stress (1.01 vs. 0.48), but the reduced expression of *PRLR* was not altered by herbal supplementation (0.48 vs. 0.61). Further, the temperature x diet interaction did not significantly influence the expression pattern of *PRLR*.

#### 3.5.4. Expression Pattern of THRA

**Figure 6:**
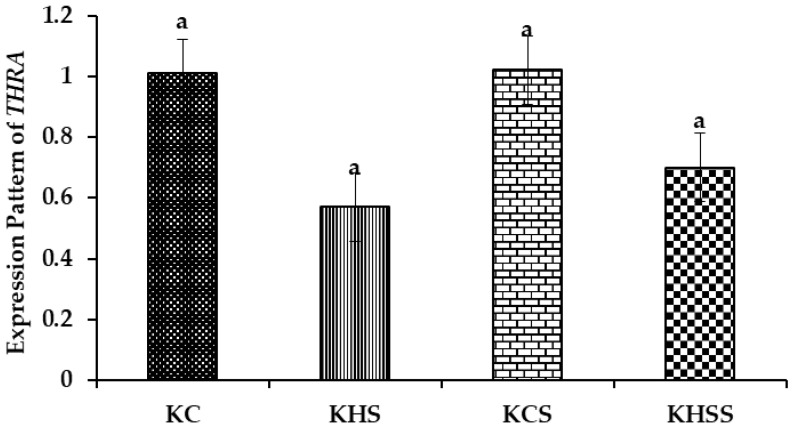
Effect of heat stress and herbal supplementation on the THRA mRNA expression pattern in Kenguri sheep. The expression patterns of *THRA* in the KC, KHS, KCS, and KHSS groups were 1.01 ± 0.10, 0.57 ± 0.10, 1.02 ± 0.10, and 0.70 ± 0.10, respectively. KC-Kenguri Control; KHS-Kenguri Heat Stress; KCS-Kenguri Control Supplement; KHSS- Kenguri Heat Stress Supplement. Different superscript letters (a, b and c) indicated extremely significant statistical differences among the treatment groups (*p* < 0.001).

The effect of heat stress and herbal supplementation on the *thyroid hormone receptor alpha* (*THRA*) expression pattern in Kenguri sheep is described in Figure 6.

Among the different groups, on comparison, the expression pattern of *THRA* did not differ significantly. Further, the treatment, diet, and treatment x diet interaction also did not influence *THRA*, such that the expression values remained comparable across the groups.

## 4. Discussion

The results obtained from this study assessed the various phenotypic and genotypic traits governing growth performance in Kenguri sheep during heat stress exposure. This study is the need of the hour as global researchers try to find solutions for climate change associated with livestock production by identifying various climate-resilient livestock breeds. This approach may help the farming community to capitalise on such breeds by propagating them to poor and marginal farmers for sustainable livestock production. Although indigenous in nature, the available literature clearly suggests that, even among indigenous breeds, there are differences in their climate-resilient potential [40]. The present study is one such attempt to study in detail the various mechanisms that govern growth performance during heat stress exposure in Kenguri sheep. The results obtained from the study also assessed novel herbal supplements to improve the climate resilience in Kenguri sheep, which may revolutionise future breeding strategies in sheep production.

### 4.1. Temperature–Humidity Index

The THI recorded during the study period clearly demonstrates that the KHS and KHSS groups were subjected to extremely severe heat stress, while the KC and KCS animals were subjected to comfort conditions. This was in accordance with the THI model developed by McDowell in 1972 [37]. This proved the hypothesis of providing a simulated condition to compare the animals in thermoneutral zone versus extreme heat stress conditions with and without herbal supplements, to explore the possibility of identifying potential phenotypic and genotypic biomarkers. The average THIs during the time duration between 10:00 h and 16:00 h in the TNZ and heating chambers were 74.38 and 74.45, respectively, which do not fall under the stress category, as per McDowell [37].

### 4.2. Feed Intake

Heat stress significantly increased the feed intake, which was not surprising given the fact that Kenguri sheep are extremely adapted and well-known for grazing during the summer months. This finding could be attributed to the indigenous nature of this particular breed. The indigenous breeds are well-known for grazing during the summer months without compromising their feed intake. Since Kenguri is an indigenous sheep breed, it could have possessed the ability to cope with heat stress without compromising feed intake. This could have been the reason for the increased feed intake, probably to supply regular energy for vital body organ functioning to dissipate body heat to cope with heat stress. However, a contrasting finding of a reduction in feed intake during extreme heat stress exposure was recorded, with the probable mechanism of reducing metabolic heat production in ruminants [41]. A study reported that heat stress reduced feed intake by stimulating the peripheral thermal receptor for transmitting a suppressive nerve impulse to the appetite centre located in the hypothalamus. The heat stress-associated reduction in feed intake could be an adaptive mechanism of the animal to generate low body heat [9]. There are also contrasting reports of recording reduced feed intake after heat stress exposure in Dorper × Pelibuey ewe lambs and Lacaune ewes [42,43]. A similar study in small-tailed Han sheep [44] reported reduced feed intake during an increased THI in ewes. Further, in the current study, the herbal supplement increased the feed intake, which could be attributed to its heat stress-protective effects to stimulate feed intake. A study conducted on Barbari kids [23] established increased voluntary feed intake after herbal mixture (Amla, Haldi, Arni, and Tulsi) supplementation. The already increased feed intake in this particular breed during heat stress exposure was further boosted by herb supplements, establishing the potential for its improved climate resilience. The significant temperature × day interaction indicates that the increased feed intake during heat stress exposure persisted throughout the study period. Further, the significant interaction between diet × day indicates that the herbal diet had a positive influence on feed intake throughout the study period. In addition, the significant interaction between temperature × diet × day clearly suggests that both heat stress and herbal feed supplements induced increased feed intake throughout the study period.

### 4.3. Water Intake

Heat stress significantly (*p* < 0.001) increased the water intake, while the herbal supplement did not alter the heat stress-induced increase in water intake. A similar study conducted in climate chambers in Malpura ewes reported significantly higher water intake in heat-stressed animals [45]. These authors attributed this to the animals meeting the increment of water requirements for heat dissipation through evaporation. In a similar study, ref. [43], it was reported that heat-stressed dairy ewes consumed more water than the control group ewes. Likewise, ref. [46] reported similar findings of heat stress associated with increased water intake in Sardinian ewes. However, ref. [47] conducted a study to compare Merino wethers and Awassi rams and reported that the water intake increased only in Awassi rams during heat stress exposure, but it remained unaltered in Merino wethers. A study conducted in Dorper × Katahdin male lambs reported a 49% higher water intake in heat-stressed lambs than their counterpart. These authors opined that increased water intake is an important thermoregulatory mechanism to avoid hyperthermia and dehydration in animals experiencing heat stress [48]. Further, it was expected that the evaporation of water content increases during heat stress and this should have influenced the water intake of the KHS and KHSS groups. Therefore, the accuracy of water intake would be determined by the rate of evaporation of water in the heat-stressed animals. The significant temperature × day interaction on water intake indicates that the severity of heat stress persisted, and Kenguri ewes increased their water intake to cope with heat stress throughout the study period.

### 4.4. Body Weight

In this study, the temperature treatment did not influence the body weight. Although exposed to such extreme heat stress, Kenguri sheep showed high resilience by maintaining their body weight. This could be attributed to the indigenous, adapted nature of this breed. The reason for no effect of increased feed intake on body weight gain during heat stress exposure could be due to the possibility of energy getting channelled only towards adaptive pathways and not towards productive pathways in these animals. However, in a study conducted on Comisanas and Sardinian sheep by [49], it was reported that their body weight reduced during heat stress exposure. Similarly, studies have noted that exposure of sheep to elevated temperatures results in a decrease in body weight [9,45]. A study conducted in Deccani and Nellore sheep also reported a significant reduction in body weight [50]. These authors opined that the combined effects of reduced feed consumption, increased energy cost required for heat dissipation, and altered metabolic and gut physiological processes could be the cause for the reduction in weight gain.

Comparing these findings, it is evident that Kenguri ewes were superior in terms of maintaining body weight during heat stress exposure. Although the feed intake increased during heat stress, it still could not reflect on body weight as the energy could have been diverted to adaptive pathways rather than production. However, as the experiment progressed, the herbal supplement was able to increase the body weight, as reflected by the significant diet x day interaction, indicating the beneficial role of herbal supplements. Likewise, in a similar study, the inclusion of herbal supplements as a mixture composed of different herbs may have had multiple effects, and it demonstrated the synergistic effect of a herbal formula in improving body weight gain in fattening lambs [8]. Similarly, a study investigated the effects of *Morinda citrifolia* (Noni) on growth and feed efficiency in large, domestic ruminants. The improvement in growth was attributed to improved immune function in Noni-supplemented calves. Further, it was suggested that the antioxidant activity could be responsible for the improved growth and feed efficiency of Noni-fed cattle [51]. The significant effect of diet x day interaction on body weight reflects the positive impact of the herbal feed additive on this particular variable throughout the study period. These findings highlight the effectiveness of herbal supplements in reducing the negative impact of heat stress on the growth performance of Kenguri sheep, thereby indicating its potential for sustainable sheep production in the changing climate scenario.

### 4.5. Allometric Measurements

Body measurements are used to evaluate the characteristics of animals that may vary due to the influence of breed evolution, environment, and nutrition. The body measurements are related to growth and can be used to predict growth [52,53]. However, neither temperature treatment nor the diet influenced any of the allometric measurements in Kenguri ewes in the present study. Similar to this finding, in another study conducted in native sheep of Bangladesh, heat exposure resulted in no significant differences in body weight, length, and heart girth [54]. However, ref. [55] reported reduced body measurements like body length, wither height, and chest girth in Naeemi sheep during the summer season, and it attributed this to the heat stress experienced by these animals. Further, the herbal supplement also did not influence the body measurements in the present study. The significant effect of diet x day interaction on body length reflects the positive impact of the herbal feed additive on this particular variable throughout the study period.

### 4.6. Endocrine Variables

#### 4.6.1. Plasma GH and IGF-1 Concentration

In the present study, heat stress did not influence the plasma GH concentration. However, the herbal supplement significantly increased its concentration during the entire study period. GH is considered one of the prominent hormones that are altered under heat stress conditions, and its plasma levels can be a potential indicator of the physiological changes in heat-stressed animals [56]. Further, it has also been established that prolonged heat exposure may decrease the GH concentration [57,58]. The non-significant effect of heat stress in the current study could be due to the extreme adaptive nature of the particular breed. Further, the non-significant effect of heat stress on the body weight in the current study establishes the climate-resilient potential of this particular breed. Similar to our finding, ref. [59] also established a non-significant effect of prolonged heat stress on blood GH concentration in sheep. The non-significant effect of heat stress on the plasma GH concentration could be partially dependent on the plane of nutrition in these animals as these animals are ad libitum fed, which should have provided them with the appropriate nutrition to counter the heat stress. Supporting this notion, the KHSS group’s animals were subjected to herbal supplements, and the concentration of GH was markedly increased in this study. Further, ref. [60] also supported this explanation through their finding of chronic heat stress mediating reduced circulating GH levels in dairy cows, and they attributed this decrease partially to the poor plane of nutrition. Similar to the results obtained for the GH level, the concentration of IGF-1 also did not differ for the heat stress treatment. Thus, the unaltered levels of GH and IGF-1 in heat-stressed Kenguri ewes demonstrate the adaptive potential of this particular breed. Further, both IGF-1 and GH were established to work in coordination in hormone response pathways controlling energy metabolism during the thermoregulation process in cattle [61]. Further, there are reports that suggest that the adverse impact of heat stress could be alleviated by regulation of IGF-1 through its thermo-protective action [62,63]. However, a study in Malpura lambs by [64] reported that heat stress significantly increased both GH and IGF-1 concentrations. In addition, the progressive increase in the GH and IGF-1 concentrations in the KHSS group as the experimental days progressed clearly demonstrates the climate-resilient potential of this particular breed, as once they adapted to the heat stress, the herbal supplement had a beneficial role by improving the GH and IGF-1 concentrations. The non-significant interaction between temperature treatment and experimental days on plasma GH and IGF-1 concentrations indicates that the heat stress did not alter the levels of these two hormones throughout the study period. However, the significant interaction between diet and experimental days clearly indicates the positive effects of the herbal feed additives on both the GH and IGF-1 concentrations persisted throughout the study period. These results clearly demonstrate the significance of using herbal supplements in ameliorating the adverse heat stress impacts on growth performance in heat-stressed sheep.

#### 4.6.2. Plasma Thyroid Hormone Concentration

Thyroid hormones have an essential role in thermoregulation and homothermy. There are also seasonal influences established on the activity of the thyroid gland and on blood thyroid hormone concentration [65]. In the present study, heat stress significantly reduced both T3 and T4 concentrations. This could be attributed to the metabolic adaptation of Kenguri ewes, reducing their levels to produce less metabolic heat to cope with the external harsh environmental condition. Similar to our findings, ref. [66] also reported reduced T3 and T4 concentrations in Iranian fat-tailed sheep. Further, ref. [67] also reported decreased blood T3 and T4 levels in indigenous sheep breeds during heat stress exposure. In addition, the herbal supplements during heat stress conditions also significantly increased the levels of both T3 and T4, reflecting the heat stress-protecting effects of these supplements. It is to be noted that there was no literature available, especially studying the influence of multiple herbal supplements on the endocrine profile of sheep. The significant temperature × day interaction on both the plasma T3 and T4 concentrations indicates that the heat stress-induced reduced plasma thyroid hormone concentration persisted throughout the study period in Kenguri ewes, probably to adapt to heat stress challenges by reducing metabolic heat production. Further, the diet × day interaction indicates that the herbal feed additives induced the increased plasma thyroid hormone concentration throughout the study period. In addition, the significant interaction between temperature × diet × day on plasma thyroid hormone concentration suggests the heat stress protection effects of herbal feed additives in heat-stressed Kenguri ewes persisted throughout the study period. These results clearly demonstrate the practical significance of herbal supplements in mitigating the adverse effects of heat stress on the growth performance of sheep.

### 4.7. Gene Expression Patterns

#### 4.7.1. The GHR and IGF-1 Expression Patterns

The *growth hormone receptor* (*GHR*) and *insulin-like growth factor* (*IGF1*) expression patterns were significantly lower during heat stress exposure. Further, the herbal supplement significantly upregulated the expression patterns of *GHR* and *IGF1*. The supplementation in the thermoneutral animals did not bring any beneficial effect, as evident from the comparable levels of expression patterns between the KC and KCS groups. However, the significant upregulation of the supplemented group during heat stress clearly establishes the heat stress-protective effects of the supplement. A study in Osmanabadi goats observed lower expression of *IGF1* during heat stress exposure and attributed this to the lower feed intake during heat stress exposure [68]. In the current study, although heat stress mediated an increase in the feed intake, the level of *IGF1* was still lower at the mRNA level, indicating the severity of heat stress in this study, and thus the increase in the feed intake could not bring about any positive change in the body weight, allometric measurements, IGF-1, and *IGF1* mRNA expression. The direct physiological effects of GH result from the activation of extracellular GHR [69]. Further, GHR mediated the effect of GH on IGF-1, as GH–GHR binding was necessary to stimulate IGF-1 synthesis and release [70]. These hypotheses have been proved by a significant decrease in the GH and IGF-1 concentration, along with the downregulation of both *GHR* and *IGF1*, in this study. Similarly, lower expressions of *GHR* and *IGF1* in Malabari goats have been reported by [15]. Likewise, ref. [71] also reported similar findings of lower expressions of *GHR* and *IGF1* in Salem black goats. These authors attributed these downregulated *GHR* and *IGF1* during heat stress exposure to the lower nutritional status of these animals. Thus, the herbal supplement during heat stress that upregulated *GHR* and *IGF1* in the present study proved that the necessary micronutrient supplementation was essential to protect the Kenguri ewes from heat stress. These findings signify the importance of supplementing essential micronutrients to improve the climate resilience in small ruminants. The significant interaction between heat treatment and diet clearly demonstrates the positive impact of herbal feed additives on *GHR* and *IGF-1* expression patterns in heat-stressed Kenguri ewes. These molecular-level results clearly demonstrate the significant role of the herbal supplement in enhancing the growth performance of heat-stressed sheep, presenting it as a viable solution to mitigate heat stress-related economic losses for sheep farmers.

#### 4.7.2. The PRLR Expression Pattern

Heat stress downregulated the *prolactin receptor* (*PRLR),* while the herbal supplement significantly upregulated its expression pattern. It was found that *PRLR* was associated with thermo-tolerance in ruminant livestock [72]. Further, ref. [73] established a lower expression pattern of PRLR in heat-stressed domestic ruminants. These authors opined that the downregulation of *PRLR* was associated with mechanisms to downregulate some metabolic process directed to reduce heat increment. Thus, in the current study, the downregulation of *PRLR* could be attributed to the adaptive mechanism of Kenguri ewes to reduce metabolic heat production to cope with extreme external severe heat stress conditions. The herbal supplementation significantly upregulated the heat stress-mediated reduced expression of *PRLR*. This herbal supplement mediated an increase of PRLR in heat-stressed Kenguri ewes that could promote the acclimatisation process involved in the regulation of several ions, extracellular volume, and body fluid, improving animal responses related to heat dissipation during chronic heat stress exposure [74].

#### 4.7.3. The THR Expression Pattern

It has been demonstrated that the *thyroid hormone receptor* (*THR*) is considered an important thermo-tolerant gene in livestock species [72]. THR signalling is required for the normal development of *parvalbuminergic neurons* (*PBNs*) linking the thyroid hormone to cardiac and temperature regulation [75]. It has been established that heat stress significantly reduces the *THR* expression in dairy cows [76]. However, in the present study, neither heat stress nor the herbal supplement influenced the expression pattern of *thyroid hormone receptor* alpha (*THRA*). The unaltered expression pattern of *THRA* could reflect the inherent potential of the Kenguri ewes to keep intact their level of mediating effective thermoregulation. The non-significant effect of both treatment and diet could reflect the better nutritional status of the Kenguri ewes.

## 5. Conclusions

This study establishes, in detail, the various impacts of heat stress on different growth performance-related variables. While the herbal supplement did not result in beneficial effects on the body weight and allometric measurements in Kenguri ewes during heat stress exposure, it did increase the plasma GH and IGF1 concentrations during heat stress exposure, reflecting its positive role on the endocrine factors controlling growth performance in these ewes. Further, the herbal supplement significantly increased the heat stress-induced reduced levels of plasma T_3_ and T_4._ In addition, heat stress significantly reduced the *GHR*, *IGF1*, and *PRLR* expression. However, herbal supplementation enhanced their expression, reflecting its positive role in the molecular mechanisms governing growth performance in Kenguri ewes. Thus, the positive impact of the herbal supplements on most of the variables studied clearly reflects their potential for improving climate resilience in Kenguri ewes. Therefore, from the study, it could be concluded that although the herbal supplements did not bring positive changes in body weight and allometric measurements, it still had a beneficial impact on the endocrinology and genes governing growth performance in Kenguri ewes. Thus, the herbal feed additive used in the study shows promise for relieving heat stress in Kenguri ewes. These results suggest further refinements are needed in identifying the active components in the herbal feed additives, and the dose used in this study should be revisited. While this study points towards the heat stress-protective effects of the herbal supplement, future research should investigate its effects on other sheep breeds in a larger population to determine its broader applicability. In addition, future work should target exploring the exact molecular mechanisms underlying its growth-protective effects and investigate its positive effects in a prolonged feeding study.

## Figures and Tables

**Table 1:** Weather variables recorded during the entire study period.

	Time of Recording	DBT (°C)	WBT (°C)	THI
Thermoneutral zone chamber	Morning (8:00)	21.78 ± 0.09	20.80 ± 0.10	71.25 ± 0.13
	Afternoon (14:00)	20.15 ± 0.05	19.19 ± 0.07	68.93 ± 0.07
Heating chamber	Morning (8:00)	22.49 ± 0.08	21.49 ± 0.08	72.27 ± 0.12
	Afternoon (14:00)	40.44 ± 0.18	32.81 ± 0.31	93.34 ± 0.34

DBT—dry bulb temperature; WBT—wet bulb temperature; THI—temperature–humidity index.

**Table 2:** Feed ingredients and chemical composition of concentrate and roughage fed to the experimental animals.

Attribute	Concentrate Mixture (kg/100 kg)	Napier Hay (*Pennisetum purpureum*)
**Ingredients**		
Maize	36	-
Wheat bran	37	-
Soybean meal	25	-
Mineral mixture	1.5	-
Salt	0.5	-
**Chemical composition (%)**		
Dry matter	90.83	92.32
Organic matter	94.02	88.59
Crude protein	23.15	6.84
Ether extract	3.77	2.72
Crude fibre	6.12	32.58
Total ash	5.98	11.41
**Fibre fractions (%)**		
Neutral detergent fibre	29.13	71.51
Acid detergent fibre	8.97	43.94
**Nutritive value**		
Total digestible nutrients (%)	71	53.20
Digestible energy (kJ/kg)	14.24	9.78
Metabolisable energy (kJ/kg)	11.78	7.98

**Table 3:** Composition of the herbal supplement.

Nutrient	Composition (100 g)
*Ocimum sanctum*	30 g
*Emblica officinalis*	30 g
*Morinda citrifolia*	20 g
*Withania somnifera*	10 g
*Phyllostachys edulis*	10 g
**Dose rate**	0.8 g/Kg BW/Day

**Table 4:** The primers used for studying the relative expression of the targeted genes.

Gene ID		Primer Sequence (5′–3′)	Product Size (bp)	Accession No.
*GAPDH*	F R	GGTGATGCTGGTGCTGAGTAG ACGATGTCCACTTTGCCAGT	147	XM_005680968.3
*GHR*	F R	AACCACCACCCAATACAG CAACGAGTACATCGGAAC	134	NM_001009323
*IGF1*	F R	AGCAGTCTTCCAACCCAA ACATCTCCAGCCTCCTCA	85	NM_001009774
*PRLR*	F R	CCCCTTGTTCTCTGCTAAACCC CTATCCGTCACCCGAGACACC	129	O46561-1
*THRA*	F R	TATCGCTGCATCACTTGCGA CTCAATCAGCTTGCGCTTGG	225	NM_001100919.3

bp—base pair; F—forward; R—reverse; *GAPDH*—glyceraldehyde-3-phosphate dehydrogenase; *GHR*—growth hormone receptor; *IGF1*—insulin-like growth factor 1; *PRLR*—prolactin receptor; *THRA*—thyroid hormone receptor alpha.

**Table 5:** Effect of heat stress and herbal supplementation on the behavioural responses in Kenguri ewes.

Variable	Temperature (T)	Diet	Day					Significance
			0	15	30	45	60	
**Feed intake** **(g/w0.75/day)**	Thermoneutral	Control	50.5 ± 0.38	47.3 ± 1.33	49.1 ± 1.24	49.1 ± 1.17	50.3 ± 0.91	Temperature.Day (*p* < 0.001); Diet.Day (*p* < 0.01), Temperature.Diet.Day (*p* < 0.01)
		Herb	51.9 ± 0.38	48.4 ± 1.33	51.5 ± 1.24	49.8 ± 1.17	51.4 ± 0.91	
	Heat stress	Control	50.9 ± 0.38	50.8 ± 1.33	51.7 ± 1.24	49.1 ± 1.17	51.4 ± 0.91	
		Herb	49.1 ± 0.38	49.3 ± 1.33	54.0 ± 1.24	54.1 ± 1.17	52.6 ± 0.91	
		Day effect	50.61 ± 0.19	48.95 ± 0.65	51.55 ± 0.59	50.54 ± 0.60	51.43 ± 0.39	Day (*p* < 0.01)
		Diet effect	50.03 vs. 51.20					Diet (*p* < 0.05)
		Temperature effect	49.93 vs. 51.30					Temperature (*p* < 0.01)
**Water intake** **(L/Kg DMI/day)**	Thermoneutral	Control	3.33 ± 0.61	5.00 ± 0.55	4.32 ± 0.49	4.23 ± 0.57	3.94 ± 0.47	Temperature.Day (*p* < 0.001)
		Herb	3.50 ± 0.61	4.67 ± 0.55	4.67 ± 0.49	5.61 ± 0.57	4.98 ± 0.47	
	Heat stress	Control	3.47 ± 0.61	7.57 ± 0.55	7.28 ± 0.49	7.49 ± 0.57	6.60 ± 0.47	
		Herb	3.46 ± 0.61	7.27 ± 0.55	7.31 ± 0.49	7.33 ± 0.57	7.29 ± 0.47	
		Day effect	3.44 ± 0.30	6.13 ± 0.27	5.95 ± 0.25	6.17 ± 0.29	5.70 ± 0.24	Day (*p* < 0.001)
		Diet effect	5.32 vs. 5.63					NS
		Temperature effect	4.45 vs. 6.51					Temperature (*p* < 0.001)

NS—non-significant.

**Table 6:** Effect of heat stress and herbal supplementation on the body weight in Kenguri ewes.

**Variable**	**Temperature (T)**	**Diet**	**Day**					**Significance**
			**0**	**15**	**30**	**45**	**60**	Diet.Day (*p* < 0.01)
Body weight (Kg)	Thermoneutral	Control	18.8 ± 0.90	19.5 ± 0.80	20.0 ± 0.79	21.0 ± 0.80	22.2 ± 0.82	
		Herb	19.1 ± 0.90	20.4 ± 0.80	21.4 ± 0.79	22.2 ± 0.80	23.7 ± 0.82	
	Heat stress	Control	19.2 ± 0.90	20.4 ± 0.80	20.6 ± 0.79	22.1 ± 0.80	22.6 ± 0.82	
		Herb	18.8 ± 0.90	19.7 ± 0.80	21.1 ± 0.79	21.7 ± 0.80	23.1 ± 0.82	
		Day effect	18.97 ± 0.45	19.98 ± 0.41	20.80 ± 0.40	21.75 ± 0.40	22.90 ± 0.42	Day (*p* < 0.001)
		Diet effect	20.64 vs. 21.12					NS
		Temperature effect	20.83 vs. 20.93					NS

NS—non-significant.

**Table 7:** Effect of heat stress and herbal supplementation on the allometric measurements in Kenguri ewes.

Variable	Temperature (T)	Diet	Day					Significance
			0	15	30	45	60	
Body Length (cm)	Thermoneutral	Control	61.2 ± 2.25	57.8 ± 1.97	63.3 ± 2.13	61.8 ± 1.40	62.5 ± 1.39	Diet.Day (*p* < 0.05)
		Herb	60.2 ± 2.25	63.0 ± 1.97	63.2 ± 2.13	61.8 ± 1.40	62.5 ± 1.39	
	Heat stress	Control	60.7 ± 2.25	58.3 ± 1.97	63.5 ± 2.13	64.5 ± 1.40	65.2 ± 1.39	
		Herb	63.8 ± 2.25	59.0 ± 1.97	61.2 ± 2.13	61.7 ± 1.40	62.0 ± 1.39	
		Day effect	61.46 ± 1.13	59.54 ± 0.99	62.80 ± 1.07	62.46 ± 0.70	63.04 ± 0.70	Day (*p* < 0.001)
		Diet effect	61.88 vs. 61.83					NS
		Temperature effect	61.73 vs. 61.98					NS
Body Height (cm)	Thermoneutral	Control	60.7 ± 2.11	64.0 ± 1.67	63.3 ± 1.56	64.3 ± 1.00	64.0 ± 1.00	NS
		Herb	59.3 ± 2.11	63.2 ± 1.67	63.2 ± 1.56	64.7 ± 1.00	64.8 ± 1.00	
	Heat stress	Control	62.0 ± 2.11	65.0 ± 1.67	65.2 ± 1.56	64.7 ± 1.00	64.8 ± 1.00	
		Herb	61.3 ± 2.11	66.0 ± 1.67	63.0 ± 1.56	64.5 ± 1.00	64.7 ± 1.00	
		Day effect	60.83 ± 1.06	64.54 ± 0.83	63.29 ± 0.78	64.54 ± 0.50	64.58 ± 0.50	Day (*p* < 0.01)
		Diet effect	63.80 vs. 63.32					NS
		Temperature effect	63.0 vs. 64.12					NS
Chest Girth (cm)	Thermoneutral	Control	67.7 ± 1.89	66.8 ± 1.84	67.3 ± 1.79	68.7 ± 1.73	69.2 ± 1.82	NS
		Herb	69.7 ± 1.89	68.7 ± 1.84	68.3 ± 1.79	70.0 ± 1.73	70.0 ± 1.82	
	Heat stress	Control	68.8 ± 1.89	68.2 ± 1.84	69.3 ± 1.79	69.5 ± 1.73	70.2 ± 1.82	
		Herb	64.2 ± 1.89	68.7 ± 1.84	66.0 ± 1.79	67.5 ± 1.73	68.2 ± 1.82	
		Day effect	67.58 ± 0.95	67.17 ± 0.92	67.75 ± 0.89	68.92 ± 0.87	69.34 ± 0.91	Day (*p* < 0.001)
		Diet effect	68.57 vs. 67.75					NS
		Temperature effect	68.63 vs. 67.68					NS

NS—non-significance.

**Table 8:** Effect of heat stress and herbal supplementation on the endocrine profile in Kenguri ewes.

Variable	Temperature (T)	Diet	Day					Significance
			0	15	30	45	60	
GH (ng/mL)	Thermoneutral	Control	2.70 ± 0.65	3.07 ± 0.48	2.82 ± 0.53	2.51 ± 0.47	2.74 ± 0.47	Diet.Day (*p* < 0.001)
		Herb	2.90 ± 0.65	2.76 ± 0.48	3.71 ± 0.53	4.41 ± 0.47	5.14 ± 0.47	
	Heat stress	Control	2.74 ± 0.65	1.97 ± 0.48	2.07 ± 0.53	1.43 ± 0.47	1.17 ± 0.47	
		Herb	2.81 ± 0.65	2.87 ± 0.48	3.94 ± 0.53	4.54 ± 0.47	4.96 ± 0.47	
		Day effect	2.79 ± 0.33	2.67 ± 0.24	3.13 ± 0.26	3.22 ± 0.23	3.50 ± 0.24	Day (*p* < 0.001)
		Diet effect	2.32 vs. 3.80					Diet (*p* < 0.01)
		Temperature effect	3.28 vs. 2.85					NS
IGF-1 (ng/mL)	Thermoneutral	Control	48.8 ± 6.47	46.5 ± 6.81	59.0 ± 5.24	52.8 ± 5.32	44.8 ± 5.46	Diet.Day (*p* < 0.001)
		Herb	49.7 ± 6.47	49.7 ± 6.81	62.1 ± 5.24	66.2 ± 5.32	73.6 ± 5.46	
	Heat stress	Control	47.1 ± 6.47	42.2 ± 6.81	39.7 ± 5.24	40.8 ± 5.32	34.7 ± 5.46	
		Herb	49.0 ± 6.47	51.9 ± 6.81	58.2 ± 5.24	66.0 ± 5.32	72.9 ± 5.46	
		Day effect	48.63 ± 3.25	48.71 ± 3.41	54.76 ± 2.63	56.45 ± 2.65	56.48 ± 2.72	Day (*p* < 0.01)
		Diet effect	45.63 vs. 60.38					Diet (*p* < 0.01)
		Temperature effect	55.77 vs. 50.25					NS
T3 (nmol/L)	Thermoneutral	Control	25.7 ± 1.55	27.5 ± 0.88	25.1 ± 1.09	27.5 ± 0.90	27.3 ± 0.81	Temperature.Day (*p* < 0.001); Temperature.Diet (*p* < 0.001); Diet.Day (*p* < 0.001)
		Herb	26.3 ± 1.55	29.7 ± 0.88	30.7 ± 1.09	33.5 ± 0.90	34.3 ± 0.81	
	Heat stress	Control	25.6 ± 1.55	23.3 ± 0.88	19.9 ± 1.09	14.2 ± 0.90	11.2 ± 0.81	
		Herb	25.7 ± 1.55	29.9 ± 0.88	31.6 ± 1.09	32.6 ± 0.90	33.7 ± 0.81	
		Day effect	25.84 ± 0.89	27.61 ± 0.48	26.85 ± 0.54	26.97 ± 0.51	26.62 ± 0.56	NS
		Diet effect	22.74 vs. 30.82					Diet (*p* < 0.001)
		Temperature effect	28.77 vs. 24.79					Temperature (*p* < 0.001)
T4 (nmol/L)	Thermoneutral	Control	96.0 ± 6.33	108 ± 5.74	108 ± 5.94	112 ± 6.52	114 ± 6.88	Temperature.Diet (*p* < 0.05); Temperature.Day (*p* < 0.01); Diet.Day (*p* < 0.001); Temperature.Diet.Day (*p* < 0.01)
		Herb	98.6 ± 6.33	115 ± 5.74	129 ± 5.94	147 ± 6.52	160 ± 6.88	
	Heat stress	Control	99.8 ± 6.33	88.8 ± 5.74	72.8 ± 5.94	69.9 ± 6.52	58.7 ± 6.88	
		Herb	97.7 ± 6.33	112 ± 5.74	124 ± 5.94	141 ± 6.52	153 ± 6.88	
		Day effect	98.02 ± 3.56	105.86 ± 2.91	108.42 ± 2.91	117.62 ± 3.23	121.28 ± 3.60	Day (*p* < 0.001)
		Diet effect	92.76 vs. 127.71					Diet (*p* < 0.001)
		Temperature effect	118.78 vs. 101.70					Temperature (*p* < 0.05)

NS—non-significant.

## Data Availability

The data can be obtained on request from the corresponding author.
